# Culture Medium Supplements Derived from Human Platelet and Plasma: Cell Commitment and Proliferation Support

**DOI:** 10.3389/fbioe.2017.00066

**Published:** 2017-11-20

**Authors:** Anita Muraglia, Van Thi Nguyen, Marta Nardini, Massimo Mogni, Domenico Coviello, Beatrice Dozin, Paolo Strada, Ilaria Baldelli, Matteo Formica, Ranieri Cancedda, Maddalena Mastrogiacomo

**Affiliations:** ^1^Biorigen Srl, Genoa, Italy; ^2^Department of Experimental Medicine, University of Genoa, Genoa, Italy; ^3^Human Genetics Laboratory, E.O. Ospedali Galliera, Genoa, Italy; ^4^Clinical Epidemiology, Ospedale Policlinico San Martino, Genoa, Italy; ^5^Transfusion Center, Ospedale Policlinico San Martino, Genoa, Italy; ^6^Plastic and Reconstructive Surgery, Ospedale Policlinico San Martino, Genoa, Italy; ^7^Orthopedic, Traumatology and Vertebral Surgery, Ospedale Policlinico San Martino, Genoa, Italy

**Keywords:** platelet lysate, platelet factors, stem cell proliferation, cell therapy, cell line culture

## Abstract

Present cell culture medium supplements, in most cases based on animal sera, are not fully satisfactory especially for the *in vitro* expansion of cells intended for human cell therapy. This paper refers to (i) an heparin-free human platelet lysate (PL) devoid of serum or plasma components (v-PL) and (ii) an heparin-free human serum derived from plasma devoid of PL components (Pl-s) and to their use as single components or in combination in primary or cell line cultures. Human mesenchymal stem cells (MSC) primary cultures were obtained from adipose tissue, bone marrow, and umbilical cord. Human chondrocytes were obtained from articular cartilage biopsies. In general, MSC expanded in the presence of Pl-s alone showed a low or no proliferation in comparison to cells grown with the combination of Pl-s and v-PL. Confluent, growth-arrested cells, either human MSC or human articular chondrocytes, treated with v-PL resumed proliferation, whereas control cultures, not supplemented with v-PL, remained quiescent and did not proliferate. Interestingly, signal transduction pathways distinctive of proliferation were activated also in cells treated with v-PL in the absence of serum, when cell proliferation did not occur, indicating that v-PL could induce the cell re-entry in the cell cycle (cell commitment), but the presence of serum proteins was an absolute requirement for cell proliferation to happen. Indeed, Pl-s alone supported cell growth in constitutively activated cell lines (U-937, HeLa, HaCaT, and V-79) regardless of the co-presence of v-PL. Plasma- and plasma-derived serum were equally able to sustain cell proliferation although, for cells cultured in adhesion, the Pl-s was more efficient than the plasma from which it was derived. In conclusion, the cells expanded in the presence of the new additives maintained their differentiation potential and did not show alterations in their karyotype.

## Introduction

Present cell culture medium supplements, in most cases based on animal sera, are not fully satisfactory. The adoption of an animal-free culture medium is particularly relevant in establishing culture conditions for isolation and expansion of cells intended for clinical applications. Platelet growth factors can support cell proliferation and differentiation and human platelet derivatives were proposed as tissue culture supplements alternative to fetal calf or fetal bovine serum (FCS or FBS). Platelet derivatives used as cell culture medium supplements, are commonly provided in the form of platelet lysate (PL) within a small amount of plasma.

Plasma is the physiological fluid of blood and serum is the fluid remaining from plasma after fibrinogen, prothrombin, and other clotting factors have been removed. *In vivo*, cells are normally exposed to plasma and come in contact with serum only at the time of clot formation following an injury. Though, the use of plasma in cell culture presents some problems. Citrate, a calcium chelator, is the standard plasma anticoagulant used in the process of blood and plasma collection, but fibrin clots may still form when plasma is added to culture media, which contain calcium (Ayache et al., 2006). This is often prevented by adding heparin to the PL (Kocaoemer et al., 2007). However, heparins are active factors that bind growth factors and may interfere with cell growth (Vannucchi et al., 1990; Tiozzo et al., 1991; Cavari et al., 1993; Khorana et al., 2003). Heparin negatively affected proliferation and motility of vascular smooth muscle cells (Mishra-Gorur and Castellot, 1999; Gilotti et al., 2014) and inhibited growth of osteoblasts and MSC under conventional culture conditions (Andress, 1995; Handschin et al., 2005; Papathanasopoulos et al., 2011). It was also shown that a relatively high concentration of heparin in culture media supplemented with human PL impaired adipogenic and osteogenic differentiation of mesenchymal stem cells (MSC) (Hemeda et al., 2013). Others reports showed that heparin interfered with the functional capacity for migration and homing of BM-derived mononuclear cells used in cardiovascular repair (Seeger et al., 2012). Moreover, commercially available heparin is manufactured primarily from porcine sources and, being of animal origin, it represents a limit in the development of a totally xeno-free medium. Although porcine heparin is approved for human use, there are examples of hypersensitivity to the molecule (Bottio et al., 2003; Huang et al., 2012).

To overcome the need of heparin to prevent clotting after PL addition to the culture medium, different options, including the use of serum, were proposed. In principle, a human serum could be obtained by letting fresh whole blood, collected without any anticoagulant, to clot several hours before high-speed centrifugation. Being deprived of coagulation factors, this serum, containing also platelet factors, can be added to the cell culture medium without that fibrin clots may form. However, this way one can obtain only small aliquots of PL, mainly for research use. In fact, this type of strategy does not allow the preparation of large batches of standardized, quality controlled PL starting from outdated blood donations. Indeed, an adopted approach is the production of a serum-converted PL from pooled platelet-rich plasma (PRP) derived from buffy coats (BC), i.e., fractions of blood which are by-products of plasma preparations routinely performed in the Blood Banks. The plasma-coagulation step is obtained by the addition of calcium chloride and/or thrombin (in most cases of animal origin). However, by this procedure, the relative concentration of factors before and after coagulation can vary. When the concentration levels of 100 soluble factors were measured in plasma and serum using a multiplexed ELISA assay, a comparison revealed that concentrations of two factors were higher in plasma, whereas the concentrations of 18 factors, including 11 chemokines, were higher in serum (Ayache et al., 2006). Conflicting results exist in the literature with regard to the comparison of the biological activities of plasma and its derived serum. Mojica-Henshaw et al. (2013) reported that PL-serum was less efficient than the sister counterpart PL-plasma in supporting MSC proliferation although both lysates supported the cell tri-lineage differentiation potential. A beneficial effect of the fibrinogen-depleted lysate was instead observed with regard to the immunosuppressive properties of MSC (Copland et al., 2013).

An additional possibility to avoid the use of heparin is the production of a PL devoid of plasma through repeated cycles of platelet washing with a saline solution prior their rupture and release of bioactive factors. This PL sustained cell proliferation, comparable to FBS, in short-term (1–7 days) cultures of renal epithelial cells, of either animal or human origin, in adhesion and of human lymphoblastoid cells in suspension (Rauch et al., 2011). The mitogenic effect induced by the PL addition was visualized also by the activation of the ERK1/2 factors. However, the sustainability of long-term cell expansion in the presence of PL obtained from washed platelets was not investigated and no experimental evidence was given in the published reports of a long-term cell culture in the continuous presence of a plasma-free or serum-free PL as the only medium supplement.

As mentioned above, serum is usually obtained by allowing a whole blood specimen to clot prior to centrifugation. The first studies, using human serum obtained by conventional blood coagulation as cell culture supplement, showed the efficient isolation and expansion of bone marrow MSC that maintained their osteo-adipogenic differentiation potential (osteogenic differentiation was higher in autologous serum rather than in FBS) (Stute et al., 2004). Furthermore, bone-marrow-derived MSC expanded in the presence of autologous human serum from whole blood presented a higher cell motility compared with the ones expanded in the presence of FCS (Kobayashi et al., 2005). Autologous serum was shown to be a suitable supplement also for the *in vitro* expansion of dental pulp stem cells without altering their multi-lineage differentiation ability (Pisciotta et al., 2012). In some cases, serum was also successfully derived by the clotting of umbilical cord whole blood. Human MSC from bone marrow and umbilical cord, isolated and expanded in allogenic cord blood serum (CBS) displayed higher self-renewal and a delayed senescence compared to cells cultured in fetal bovine serum (Shetty et al., 2007). Moreover, MSC cultured in the presence of CBS showed an enhanced and accelerated osteogenic differentiation and a repressed adipogenic differentiation (Jung et al., 2009). Off the clot AB serum is commercially available and was successfully used for isolation and expansion of cells, such as bone marrow MSC and hematopoietic stem cells (Anselme et al., 2002; Yamaguchi et al., 2002). Allogenic human AB-serum was successfully used also for adipose MSC long-term culture (Kocaoemer et al., 2007). Contradictory results, however, have been reported on the use of allogeneic human serum (Shahdadfar et al., 2005; Le Blanc et al., 2007; Tateishi et al., 2008; Turnovcova et al., 2009).

Alternatively, serum can be derived from blood plasma that has been treated with anticoagulants and from which blood cells, including red blood cells, white blood cells, and platelets, were removed by centrifugation [platelet-poor plasma (PPP)] or by plasma directly collected by apheresis. Also in this case, coagulation is obtained by addition of calcium cations and/or thrombin treatment. However, depending on the protocols to obtain the PPP, preparations may contain residual platelets and, when present, these residual platelets are activated during the centrifugation steps and the coagulation process and undergo a degranulation of the alpha granules, resulting in the release of their growth factor content. Therefore, the level of platelet growth factors in the final serum may change depending on the presence of platelets in the source material and this may significantly change the biological effect of serum when used as supplement in a cell culture medium. Tanaka et al. described a more pronounced stimulation of proliferation of human auricular chondrocytes when a serum derived from plasma, including platelets was compared to a serum derived from a plasma depleted of platelets although no significant differences were observed on the cartilage matrix deposition by chondrocytes under the different serum conditions (Tanaka et al., 2008). Recently, a comparison was performed between two different plasma sources to obtain human serum, plasma removed from blood after 24 h from collection and plasma devoid of cryoprecipitate. Serum was obtained after coagulation in the presence of calcium ions. Both forms of plasma-derived serum were effective in sustaining fetal umbilical cord matrix derived MSC proliferation as the standard supplement bovine serum (Dos Santos et al., 2017).

The different abilities of plasma and serum to modulate cell growth was investigated already in the 1970s. Initial studies indicated that cells did not proliferate in plasma containing medium, but they proliferated actively when they were exposed to serum (Balk et al., 1973). However, the initial comparison was made between platelet-free plasma and serum containing platelet mitogens. Indeed, the addition of platelets and calcium to platelet-free plasma increased the activity of the obtained plasma-serum to the same level achieved with blood serum (Ross et al., 1974). Also the tridimensional environment to which cells are exposed to is crucial in modulating cell behavior. Gospodarowicz and Ill (1980) reported that bovine vascular smooth muscle cells in Petri dishes exposed to plasma proliferated poorly compared to when exposed to serum from whole blood, but, when the same cells were seeded on ECM-coated dishes, they proliferated equally well in the presence of either plasma or serum. Since this pioneering work, taking advantage of different cell types, different substrates, and different culture conditions, other authors have investigated the abilities of plasma and serum to promote cell growth (Stute et al., 2004; Shahdadfar et al., 2005; Mizuno et al., 2006). Published results are sometimes contradictory. However some general conclusions can be made out of these publications: (i) platelet-depleted plasma or serum derived from this plasma are poor cell growth inducers in cultures of primary cells (Ross et al., 1974, 1978; Gajdusek et al., 1980); (ii) serum allows a better adhesion of the cells to the substrate than plasma, unless a coating of the culture dishes by serum or extracellular matrix proteins is adopted.

Several years ago, Rutherford and Ross (1976) reported that exposure of quiescent cells to whole blood serum or platelet-free plasma-serum plus crude platelet factors preparations stimulated cell proliferation. However, no accurate investigations were ever made to distinguish between the role played by factors and molecules released by platelets and the serum components. Aim of our experimental study was to verify the role played by PL devoid of plasma or serum contaminants (v-PL) and/or plasma or plasma-derived serum (Pl-s), used as supplements to the culture media, on the viability, proliferation kinetic and differentiation potential of MSC derived from human tissue. The effect on the proliferative capability of these blood-derived components was also tested on several cell lines. We report that, although factors and molecules released by platelets (PL in saline solution) were capable of activating the cell proliferation machinery (ERK and AKT phosphorylation, Cycline D1 induction, etc.) the PL itself used as single additive to the culture medium was unable to support cell proliferation unless the plasma or serum components were also present in the culture medium. Interesting, in cells that were constitutively stimulated, such as different cell lines of human or animal origin, or in some cultures of cells derived from fetal tissues, the addition of PL to the culture medium was not an absolute requirement and cell proliferation could be obtained by the simple addition of PL-free serum.

## Materials and Methods

### Production of Virgin-Platelet Lysate (v-PL) and Plasma-Serum (Pl-s)

**Figure 1:**
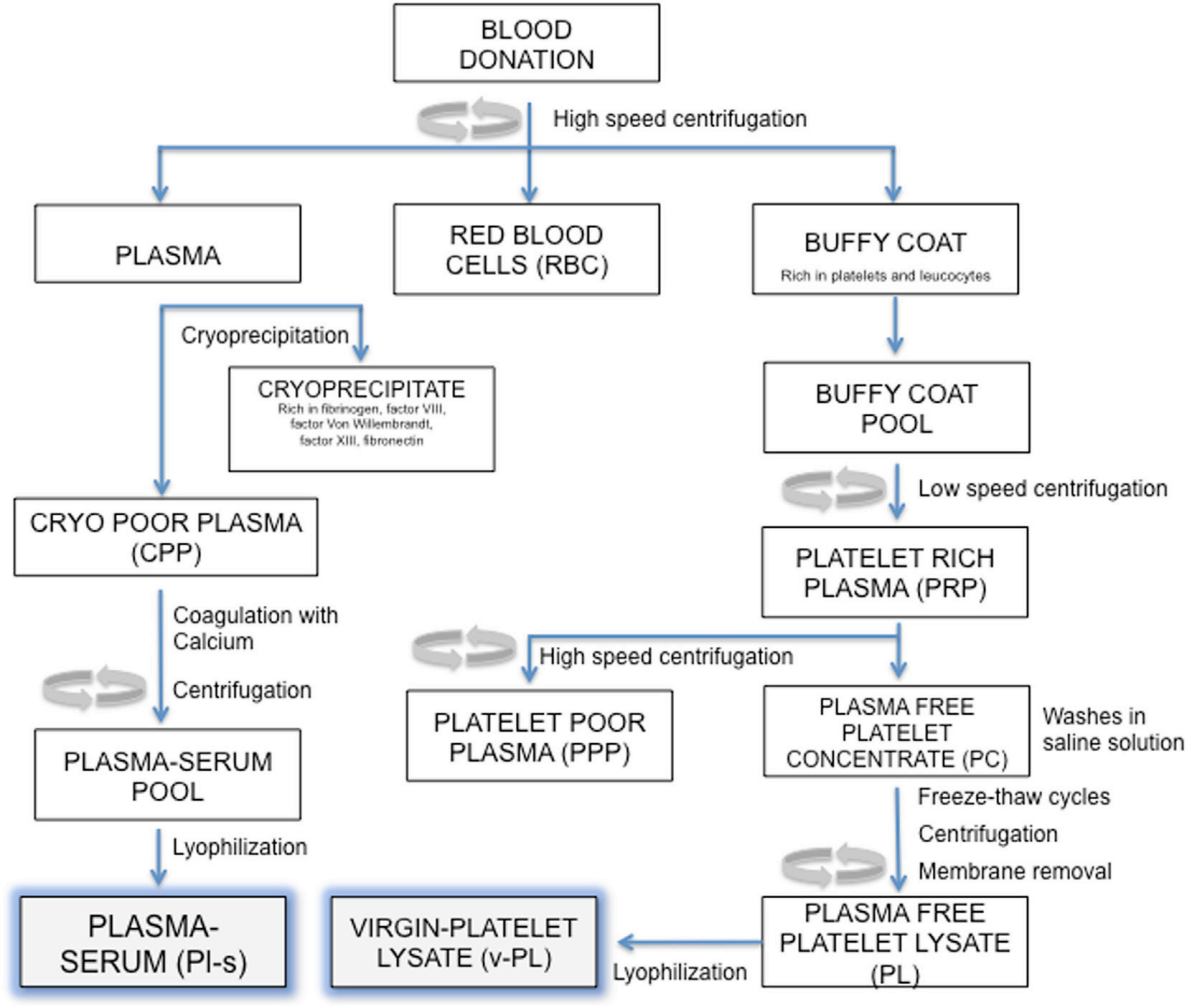
Manufacturing process outline. Steps in this procedure occur within a sterile closed system thanks to the possibility of performing sterile connections between blood bags.

An outline of the manufacturing process is reported in Figure 1. All separation steps were made within a sterile closed system. The high-speed centrifugation of a whole blood unit separates different phases: the plasma at the top, the buffy coat (BC) layer (enriched in platelets and leukocytes) at the interface and the red blood cells fraction at the bottom. For the preparation of the virgin-Platelet Lysate (v-PL), pools of BC units (not usable for transfusion purposes; up to 300 total units) were centrifuged at low speed. The PRP was recovered from the upper part of the blood bag and high-speed centrifuged to separate an upper phase, the PPP and a lower phase, the platelet concentrate. Recovered platelets were subjected to three washes in sterile saline solution. After the third wash, the platelet concentrate was suspended in saline solution and the platelet concentration adjusted to 10 × 10^6^ plt/μl. The platelet concentrate underwent three freeze–thaw cycles. A high-speed centrifugation was then performed to sediment platelet membranes and debris. The different obtained supernatants, the plasma-free platelet lysates (v-PL) were combined in a single pool before being divided into aliquots and lyophilized.

The Plasma-serum (Pl-s) was obtained from several frozen plasma units. Each plasma unit was slowly thawed at 4°C to separate the cryoprecipitate and the cryo-poor plasma (CPP). The CPP was added of calcium chloride (2 mg/ml) and then subjected to a coagulation step at 37°C up to 6 h. After the coagulation step, the blood bag was high-speed centrifuged to separate the coagulum. The liquid phase, the plasma-serum (Pl-s), was recovered, and the pool of 3–10 units was divided into aliquots and lyophilized. Pl-s and v-PL were frozen at −20°C and exposed to the freeze-drying procedure by means of the Heto LYOPRO 3000 (Analytical Control De Mori, Milano) apparatus. The lyophilization process was conducted for about 20 h until the water content was completely sublimated and the vials were closed under vacuum environment.

Both lyophilized products were stored at −20°C until use and generally used for the experiments within 3–4 months from their production.

### Cultures of Human MSC and Human Cell Lines

The use of human samples was approved by the institutional Ethics Committee of the Ospedale Policlinico San Martino, Genoa, Italy and done in accordance with the Helsinki Declaration of 1975.

Primary cultures of human MSC of different origin were established from biopsies of discarded surgical samples, lipoaspirates for cosmetic reasons, bone marrow of patients who underwent orthopedic surgery or cord blood of healthy mothers. The patient/donor-informed consent was obtained prior sample processing. Human bone marrow-derived mesenchymal stem cells (BM-MSC) were obtained from bone marrow samples and adipose-derived mesenchymal stem cells (AD-MSC) were derived from liposuction waste according to published procedures (Muraglia et al., 2015). Umbilical cord-derived MSC (UC-MSC) were kindly provided by Dr. Introna M. (AO Papa Giovanni XXIII USS Center of Cell Therapy “G. Lanzani” USC Hematology, Bergamo, Italy). Cells were isolated from cord blood tissue collected from pregnant women after either normal vaginal delivery or Cesarean sections. The UC processing was performed in accordance with the protocol for the isolation and expansion of UC-MSC as previously described (Capelli et al., 2011). Primary cultures of cells derived from adult tissues (AD-MSC, age range: 50–60 years, female; BM-MSC, age range: 33–60 years, male and female; UC-MSC, age range: 30–40 years, female. Number of cell cultures, *n*, is indicated in the legend of the figures for each cell type) were initially isolated and expanded in 10% FCS (passage 0). The experiments were performed on cells derived from different donors. At the time of the first passage, the culture medium was replaced with medium supplemented with Pl-s and v-PL in different relative ratios and the cultures continued by passaging the cells before they reached confluence for about 30 days.

Cell lines U-937 and HeLa were purchased from the Interlab Cell Line Collection of the Biological Bank and Cell Factory of the Ospedale Policlinico San Martino (Genoa, Italy). HaCaT cell line was kindly provided by Dr. Pellegrini G. (Centro di Medicina Rigenerativa “Stefano Ferrari” Modena, Italy).

Basal medium for primary cell cultures was α-MEM (Lonza, Belgium), whereas cell line basal medium was MEM (EBSS) for HeLa cells, DMEM for HaCaT, and V-79 cells and RPMI for U-937 cells (all from Euroclone, Italy). In all cases, the basal medium was supplemented with 100 IU/ml penicillin and 100 µg/ml streptomycin, 2 mM l-glutamine and, where indicated, with Pl-s and v-PL used alone or in different combinations (5% v-PL alone or 10% Pl-s or 10% Pl-s + 1% v-Pl or 10% Pl-s + 5% v-PL). In some control experiments, medium was supplemented with 10% fetal calf serum (FCS, Invitrogen, USA) instead of Pl-s and v-PL. Cells were detached from the culture dish with 0.05% trypsin and 0.01% EDTA (Euroclone, Italy). Trypsin activity was neutralized with a trypsin soybean inhibitor solution [0.5 mg/ml in phosphate buffered saline (PBS); GIBCO, USA].

### Human Articular Chondrocyte (HAC) Cultures

Femoral heads or femoral condyles were collected from patients undergoing hip or knee arthroplasty respectively, with their informed consent. The patients’ age ranged from 61 to 81 years old. All obtained samples were processed immediately after the surgery and the experiments were performed on cells derived from different donors. After washing the tissue fragment with sterile PBS, the articular cartilage was isolated using a sterile blade, minced into small pieces, and exposed to repeated cycles of digestion at 37°C in serum-free Coon’s modified Ham’s F12 medium (Biochrom, Germany) containing 0.25% (v/v) trypsin (GIBCO, USA), 1 mg/ml hyaluronidase type II (Sigma Aldrich, USA), 400 U/ml collagenase type I and 1,000 U/ml collagenase type II (Worthington Biochemical, USA). Recovered chondrocytes were expanded as adherent cells (dedifferentiated chondrocytes) up to 6 passages in complete medium (Coon’s modified Ham’s F-12 medium supplemented with 2 mM l-glutamine, 100 IU/ml penicillin, and 100 µg/ml streptomycin) and 10% FCS. When indicated, the medium was additionally supplemented with 5% v-PL. Culture media were changed twice a week. Cultures were performed at 37°C and 5% CO_2_.

In some experiments, dedifferentiated chondrocytes were transferred in suspension culture in the presence of ascorbic acid to allow the formation of a cartilage-like tissue (Zerega et al., 1999).

### Cell Proliferation Assays

#### Long-term Cell Proliferation Assay—Cumulative Doubling Number Calculation

For determining the number of doublings of BM-MSC, AD-MSC, and UC-MSC in a long-term culture, after the initial selection of the cells (passage 0) in FCS supplemented medium, at 70–80% confluence, cells were detached with trypsin/EDTA solution and replated at the density of 7 × 10^4^ cells for UC-MSC and 1 × 10^5^ cells for all the other cell types, in 60 mm Ø Petri dishes, in duplicate with medium supplemented with 10% Pl-s or 10% Pl-s + 5% v-PL or 10% Pl-s + 1% v-PL.

The human cell lines U-937 (pro-monocytic cells growing in suspension), HeLa (epithelial cells with adhesion growth), HaCaT (keratinocytes with adhesion growth), and the animal cell line V79 (hamster lung fibroblasts, also growing as adherent cells) were cultured with the same supplements used for the primary cell cultures. At each passage, cells were plated at the following densities: U-937, 200,000 cells/ml in T25 flask; HeLa, 250,000 cells/plate 60 mm Ø; HaCaT, 250,000 cells/plate 60 mm Ø; and V79, 400,000 cells/plate 60 mm Ø.

To monitor cell proliferation, the performed cumulative doubling number was calculated when the cells reached the sub-confluence at each passage and derived at different culture times (every 5 days until the end of the culture corresponding to about 30 days) as reported into the graphics. Cells were counted using a cell counting chamber under the microscope without any staining.

#### Short-term Cell Proliferation Assay

For determining cell proliferation rate during a short time period (about 1 week), BM-MSC, HeLa, and U-937 chosen as representative of primary cell cultures and cell lines growing in adhesion and in suspension, respectively, were plated for BM-MSC at 50,000 cells and for Hela, at 100,000 cells/well in 6 multi-well plates. U-937 were seeded at 100,000 cells/ml in T25 flask. The number of cells was determined at different time intervals during a week. Results were expressed as the average of at least duplicate independent experiments.

### Cell Proliferation Assay for Articular Chondrocytes—Crystal Violet Staining

Cells were seeded in 96 multi-well plates and cultured in the presence of different medium supplements as indicated. At different times of the culture, after extensive washing with PBS, cells were stained with 50 µl staining solution [0.75% (g/ml) crystal violet (Sigma-Aldrich, USA), 0.35% (g/ml) NaCl, 32.3% (v/v) absolute ethanol, 8.64% (v/v) formaldehyde 37%] for 20 min at room temperature (RT). Cells were then washed five times with water and dried by exposing the plate to air under a chemical hood. To each well 100 µl eluent solution [50% (v/v) absolute ethanol and 1% (v/v) acetic acid] were added and the absorbance at 595 nm measured within 10–30 min with a spectrophotometer AD 200 (Beckman Coulter, USA). For each experimental condition, quintuplicate assays were performed. Results were expressed as the average of at least three independent experiments.

### Cell Differentiation Assays

#### Osteogenic Differentiation

Confluent BM-MSC and AD-MSC were cultured in osteogenic differentiation medium containing 10% Pl-s, 50 µg/ml ascorbic acid, 10 mM β-glycerophosphate and 10^−7^ M dexamethasone (all from Sigma). Negative control cultures were maintained in medium containing 10% Pl-s. The medium was changed three times weekly and osteogenic stimulation took place for 3 weeks. Alizarin red S staining was performed at the end of the induction period. In some experiments (*n* = 3), parallel cell cultures were conducted in the presence of 10% FCS expanded cells, as standard additive for the cell culture medium.

#### Chondrogenic Differentiation

Dedifferentiated chondrocytes were transferred in suspension culture in 15 ml Falcon tubes to prevent cell attachment, but still ensuring cell–cell interactions, and in the presence of 100 µg/ml ascorbic acid (Sigma Aldrich, USA) to allow the organization of a cartilage matrix and the formation of a cartilage-like tissue. Cell aggregates were cultured with ascorbic acid for 10 days before being processed for histological analysis: aggregates were washed 3 times with PBS, fixed with 3.7% PFA for 20 min at 4°C and embedded in paraffin. Paraffin embedded samples were sectioned in slices of 5–6 µm thickness. Slices were adhered on Superfrost Ultra Plus Slides (Thermo Scientific, Germany) coated with poly-l-lysine (Sigma Aldrich, USA), dewaxed to remove paraffin and processed for immunohistochemistry.

Sections were permeabilized with 0.2% Triton in PBS for 10 min, treated with 4% H_2_O_2_ for 30 min at RT to inhibit endogenous peroxidase activity, rinsed with PBS 3 times × 5 min, incubated with hyaluronidase type II (Sigma Aldrich, USA) at concentration of 1 mg/ml in PBS (pH 6) for 30 min at 37°C and washed with PBS. After incubation with 10% normal goat serum in PBS for 1 h at RT to inhibit nonspecific binding, the sections were incubated at 4°C for 16 h with primary antibodies against α-collagen type II (1:100; CIIC1-Developmental Studies Hybridoma Bank, University of Iowa), washed three times with PBS and incubated for 1 h at RT with Labeled polymer HRP anti-mouse (Dako, Denmark). Sections were stained with *3*,*3*′-*Diaminobenzidine* (DAB, Enzo Life Sciences, USA) for 3–5 min, counter-stained with Mayer’s hematoxylin for 2 s, submersed in 0.1% NaHCO_3_ for 1 min, and finally mounted with Eukitt (O. Kindler GmbH, Germany). Images were acquired by a microscope Axiovert 200M (Carl Zeiss, Gottingen, Germany).

### Western Blot Analysis

Confluent dedifferentiated chondrocytes were supplemented with 5% v-PL either in the presence or in the absence of bovine serum. At different times from the addition of the supplement, cells were washed with PBS and collected for western blot analysis. Electrophoresis was performed in reducing conditions using 25 μg of protein loaded on a 4–12% NuPAGE Bis-Tris gel (Invitrogen, Italy) as described (Ulivi et al., 2006). Primary antibodies tested were α-Cyclin D1 (1:250) and α-Actin (1:200) (Santa Cruz Biotechnology, USA), α-phospho Erk1/2 (1:1,000), α-phospho Akt (1:1,000) (Cell Signaling Technology, USA). Secondary HRP conjugated anti-mouse and anti-rabbit antibodies were both diluted 1:5,000 (GE Healthcare, UK), peroxidase conjugated anti goat antibody was diluted 1:10,000 (Jackson Immunoresearch, USA). ECL was purchased from GE Healthcare, UK.

Images were scanned using the Epson perfection 1260 scanner (Epson, Italy) and band densities were quantified using the ImageJ software (http://rsbweb.nih.gov/ij/download.html). Western blots were performed on three different independent primary cultures.

### Karyotype Analysis

40,000 BM-MSC expanded in medium containing 10% Pl-s + 1% v-PL were plated in slide flasks (Thermo Fisher Scientific, Roskilde, Denmark) and cultured at 37°C in 5% CO_2_ atmosphere. The slide flasks were checked every day for cell growth. When several macroscopic colonies were visible the medium was replaced with 2.5 ml of fresh medium containing Colcemid solution (6 µg/ml; Sigma Aldrich, USA) and incubation continued. After 2 h, the medium was removed and replaced with 5 ml of hypotonic solution [tri-Sodium citrate dehydrate 17 mM/Potassium Chloride 75 mM (1:1) solution] followed by an incubation of 10 min. After that 0.5 ml of fixative ethanol/acetic acid (3:1) solution were added to the flask for 10 min at RT. The solution was then removed and replaced by 2.5 ml of fresh fixative followed by an incubation of 15 min (repeated three times).

Chromosome staining was performed by Q-banding technique (Quinacrine stain, DBA Italia, Italy). Each slide was examined and photographed under a fluorescence microscopy (Olympus) using software MACTYPE 5.4.1 (Apple). A minimum of 10 metaphases for each sample were captured and analyzed.

### Growth Factor Quantification

PDGF-BB and VEGF were quantified by an ELISA assay on Pl-s and v-PL. The adopted kits were from RayBiotech (USA) for PDGF-BB and from Invitrogen (USA) for VEGF, respectively. The assays were performed according to the manufacturer directives.

### Statistics

Differences between cell culture conditions were assessed by the non-parametric Mann–Whitney test. Statistical significance was accepted for any *p*–value <0.05. Analyses were performed using the SPSS package (version 21 for Windows, IBM).

The data were expressed in the graphs as mean ± SE.

## Results

### Growth Factor Quantification in the v-PL and the Pl-s

v-PL and Pl-s were produced according to the protocol described in the Section “Materials and Methods” and outlined in Figure 1. An ELISA assay was performed for the quantification in the two culture medium supplements of PDGF-BB and VEGF as representative indicators of platelet factors present in a relatively high and relatively low concentration, respectively (Table 1).

**Table 1:** Concentrations of PDGF-BB and VEGF in the culture medium supplements.

Sample	PDGF-BB ng/ml^a^ (mean ± SD)	VEGF ng/ml^b^ (mean ± SD)
Plasma-serum (Pl-s)	0.11 ± 0.06	0.02 ± 0.01
virgin-Platelet lysate (v-PL)	64.58 ± 9.10	4.35 ± 1.23

*^a^Average of four different preparations*.

*^b^Average of two different preparations*.

### Stimulation of Cell Proliferation by v-PL: Primary Cell Cultures versus Cell Lines

**Figure 2:**
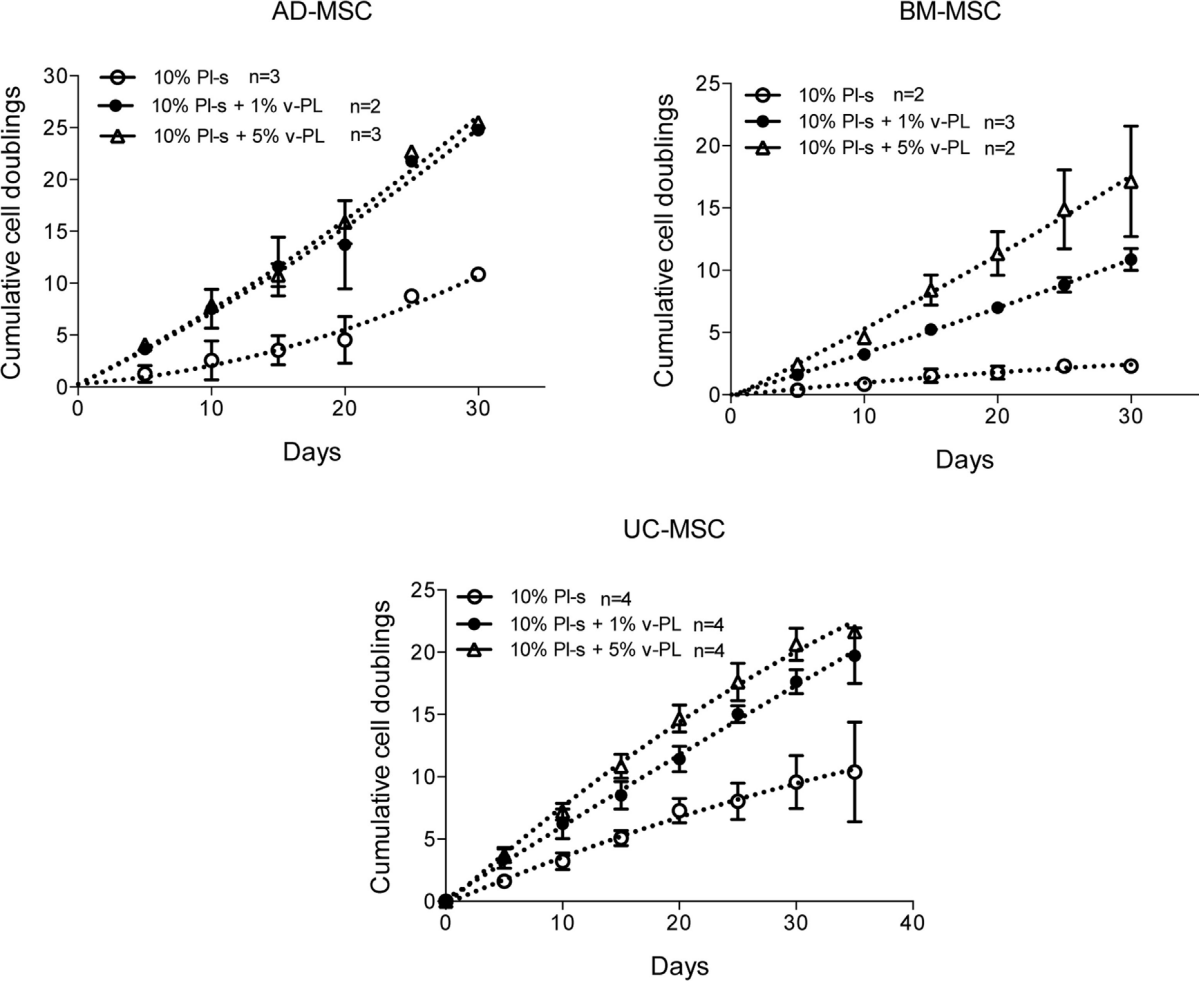
v-PL effect on cell proliferation—primary cultures. Adult tissue derived primary cells, adipose-derived mesenchymal stem cells (AD-MSC) and bone marrow-derived mesenchymal stem cells (BM-MSC) or fetal tissue derived cells, umbilical cord-derived MSC (UC-MSC) initially selected and expanded in medium supplemented with 10% FCS were transferred to media with different platelet- and plasma-derived supplements. Their proliferation rate was monitored by calculating the number of doublings performed during the time in culture. Number (*n*) of cell cultures used for the experiments is indicated on the graph for each cell type.

Mesenchymal stem cells derived from adult tissues (AD-MSC and BM-MSC) or from a fetal tissue (UC-MSC) initially isolated and expanded in 10% FCS (passage 0) were transferred in medium supplemented with Pl-s and v-PL in different relative ratios (10% Pl-s or 10% Pl-s + 1% v-Pl or 10% Pl-s + 5% v-PL) and the cultures continued by passaging the cells before they reached confluence for about 30 days. By the evaluation of the cumulative number of population doublings performed in the different culture conditions, the following conclusions were inferred: (i) In general, cells cultured with Pl-s alone showed a low or no proliferation rate in comparison to cells grown with the combination of Pl-s and v-PL; (ii) In particular, BM-MSC essentially did not proliferate in the presence of Pl-s alone (about 2 cell doublings in 30 days) whereas underwent 10.5 and 17 cell doublings in 10% Pl-s + 1% v-PL and 10% Pl-s + 5% v-PL culture medium, respectively; (iii) AD-MSC and UC-MSC performed about 10 doublings in 10% Pl-s and about 25 and 20 doublings in the presence of Pl-s and v-PL, respectively (Figure 2). For all three primary cultures, the observed difference in the proliferation rate between the cultures performed in 10%Pl-s and the cultures performed in 10%Pl-s + v-PL either 1 or 5% resulted statistically significative. No statistically significant differences were observed between 10% Pl-s + 1% v-PL or 10% Pl-s + 5% v-PL (Table 2).

**Table 2:** Statistical comparison of culture conditions as depicted in the corresponding figures.

		Culture condition							
Cell type	*p*-value	10% Pl-s	10% Pl-s + 1% v-PL	10% PL-s + 5% v-PL	10% FCS	10% FCS +5% v-PL	5% plasma +1% v-PL	5% Pl-s +1% v-PL	10% plasma
Figure 2									
AD-MSC	0.027	X	X						
AD-MSC	0.031	X		X					
AD-MSC	0.837		X	X					

BM-MSC	0.002	X	X						
BM-MSC	0.004	X		X					
BM-MSC	0.309		X	X					

UC-MSC	0.023	X	X						
UC-MSC	0.004	X		X					
UC-MSC	0.431		X	X					

Figure 3									
U-937	0.529	X	X						

HeLa	0.455	X	X						
HeLa	0.349	X		X					
HeLa	0.647		X	X					

HaCat	0.243	X	X						
HaCat	0.191	X		X					
HaCat	0.553		X	X					

V-79	0.856	X	X						
V-79	0.710	X		X					
V-79	0.443		X	X					

Figure 4									
HAC	0.078				X	X			

Figure 6									
BM-MSC	0.739						X	X	

HeLa	0.461	X							X

U-937	0.671	X							X

Figure 7									
AD-MSC	0.001		X		X				

For each cell type, the culture conditions comparatively assessed are identified by the sign “X.”

**Figure 3:**
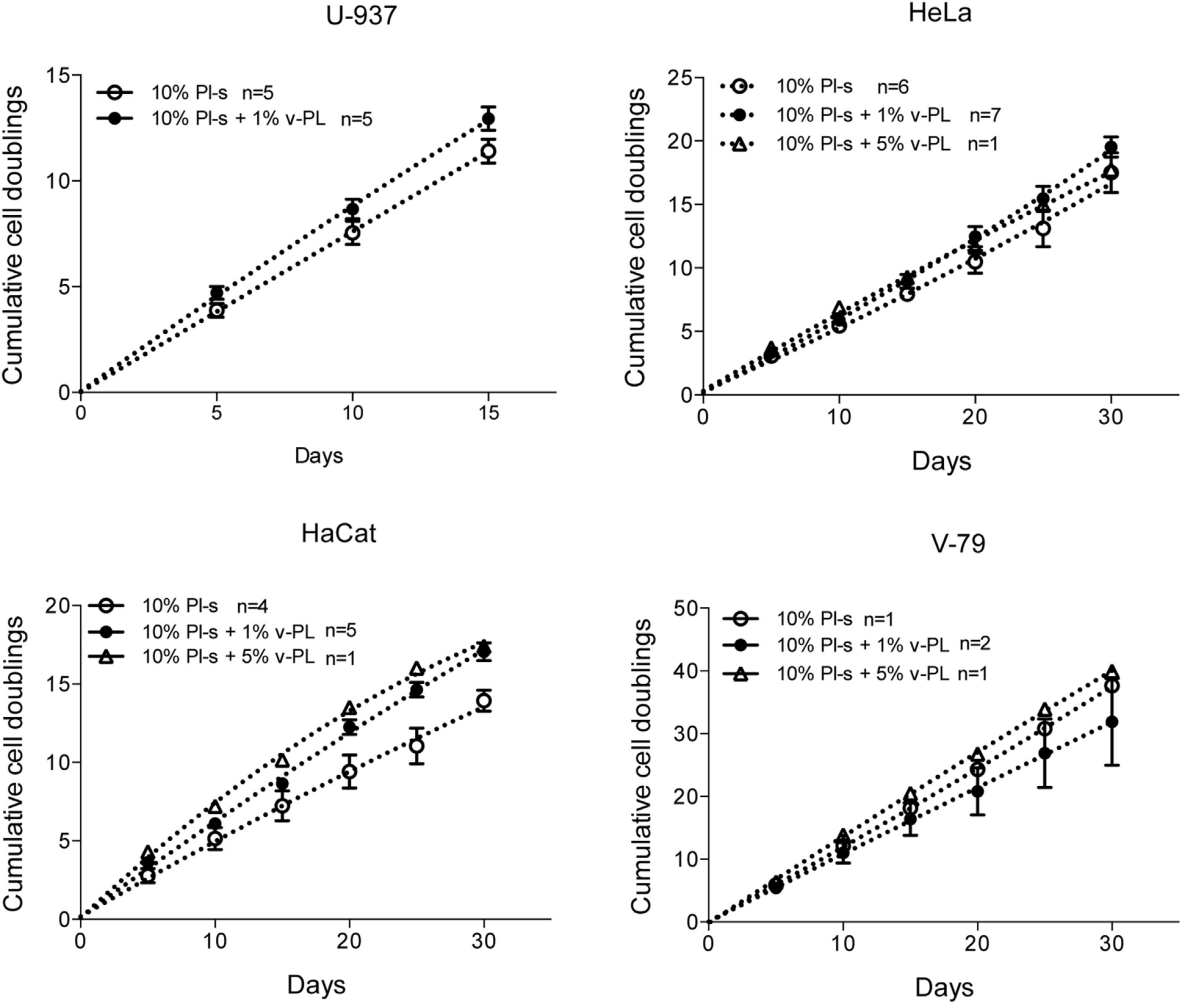
v-PL effect on cell proliferation—cell lines. The human cell lines U937, HeLa, HaCaT, and the hamster V79 cell line were cultured in the presence of different platelet- and plasma-derived supplements and their proliferation rate was monitored by calculating the number of doublings performed at different culture times. On the contrary to the behavior of the primary cell cultures, cell lines are less responsive to the v-PL mitogenic stimulus, and the culture condition in the presence of only serum is permissive to the proliferation of the cells both in adhesion or in suspension. Number (*n*) of cell cultures used for the experiments is indicated on the graph for each cell type.

The same culture medium supplements, used in the same combinations as for the primary cell cultures, were tested with human, U937, HeLa, HaCaT, and animal V-79 cell lines. Also in this case, the cell proliferation was monitored by determining the number of cell doublings at different culture times. v-PL was not required to support cell proliferation. Indeed, Pl-s alone was able to support human cell growth in a manner comparable to the different combinations of Pl-s and v-PL both for suspension (U-937) and adhesion (HeLa and HaCaT) cultures. Moreover, the human additives sustained survival and proliferation of animal derived cells (V-79, growing in adhesion) comparably to human cells (Figure 3). The statistical analysis of the different growth curves revealed no significant differences between the different culture conditions (Table 2).

### v-PL Promotes Re-Entry in the Cell Cycle of Confluent Resting Cells

**Figure 4:**
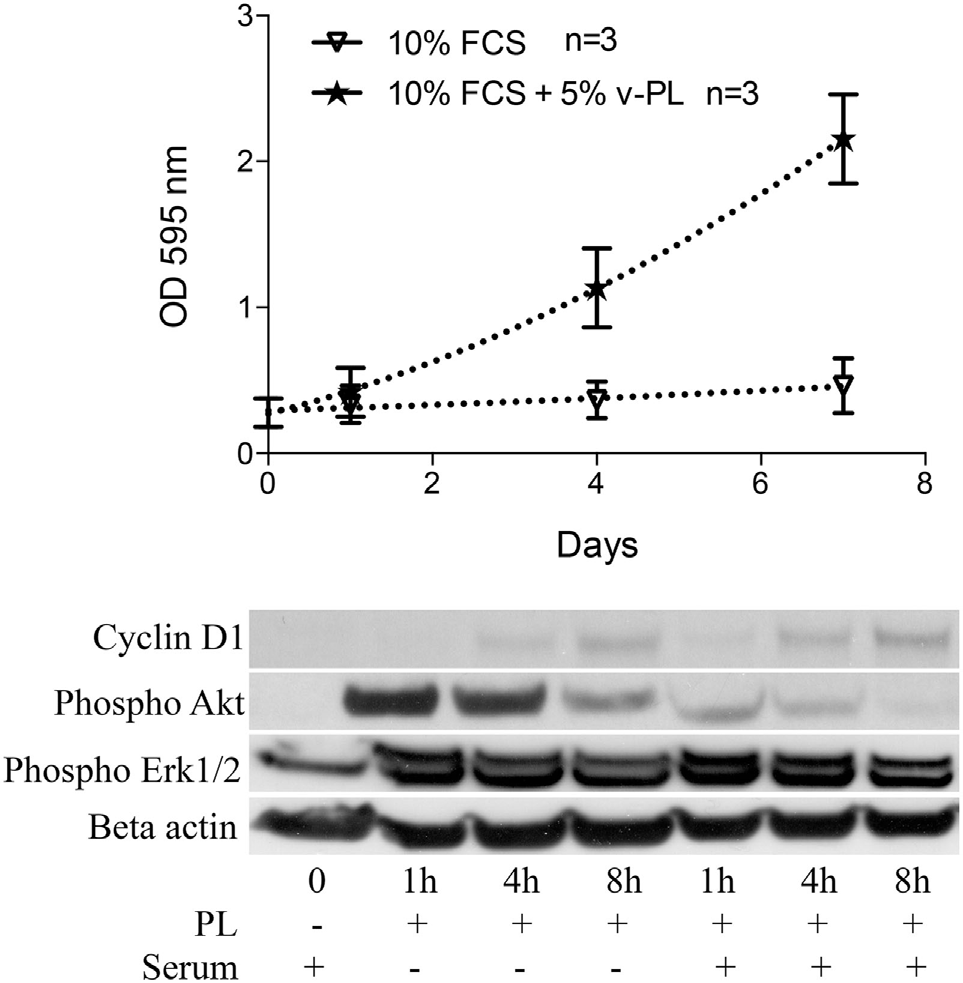
v-PL promotes re-entry in the cell cycle of confluent resting cells. Upper panel: confluent growth-arrested chondrocytes, obtained from cartilage biopsies and expanded *in vitro* in the presence of 10% FCS, were maintained in 10% serum or additionally supplemented with 5% v-PL. A crystal violet proliferation assay was performed in parallel on both cultures. Confluent cells treated with v-PL resumed proliferation, whereas the parallel control culture did not. Lower panel: Western blot analysis of proteins extracted from the cells treated with 5% PL probed with Cyclin D1, phospho Akt, phospho Erk1/2, and Actin antibodies shows that proliferation pathways were activated by v-PL also in the absence of serum. Number (*n*) of cell cultures used for the experiments is indicated on the graph for each cell type.

Confluent growth-arrested dedifferentiated HAC were maintained in the original culture medium supplemented with 10% FCS or additionally supplemented with 5% v-PL. A crystal violet proliferation assay was performed in parallel on both cultures (Figure 4 upper panel). Confluent cells treated with v-PL resumed proliferation, whereas the control culture maintained in FCS only remained quiescent and did not proliferate.

A western blot analysis of proteins extracted from control cells and cells treated with 5% v-PL for different times (1, 4, and 8 h) was performed using α-cyclin D1, α-phospho Akt, and α-phospho Erk1/2 antibodies. Actin was blotted as an internal control. After 1 h from the v-PL treatment, we observed an increase in the amount of phospho Akt and phospho extracellular signal-regulated protein kinases 1 and 2 (Erk1/2). After 8 h the activation of these proteins was almost completely over. Instead at the same time an expression of the Cyclin D1 protein reached its maximum. Interestingly, the western blot analysis also showed that these proliferation pathways were activated by PL not only in cells resuming proliferation but also in cells treated with v-PL in the absence of serum (Figure 4 bottom panel).

This confirmed and expanded our previous observation that confluent quiescent osteoblasts (Ruggiu et al., 2013) that did not proliferate when maintained in serum, once exposed to the PL mitogenic stimulus were able to activate signal transduction pathways that promoted cell growth in response to the extracellular signals.

### v-PL Can Rejuvenate a Culture of High Passage MSC

**Figure 5:**
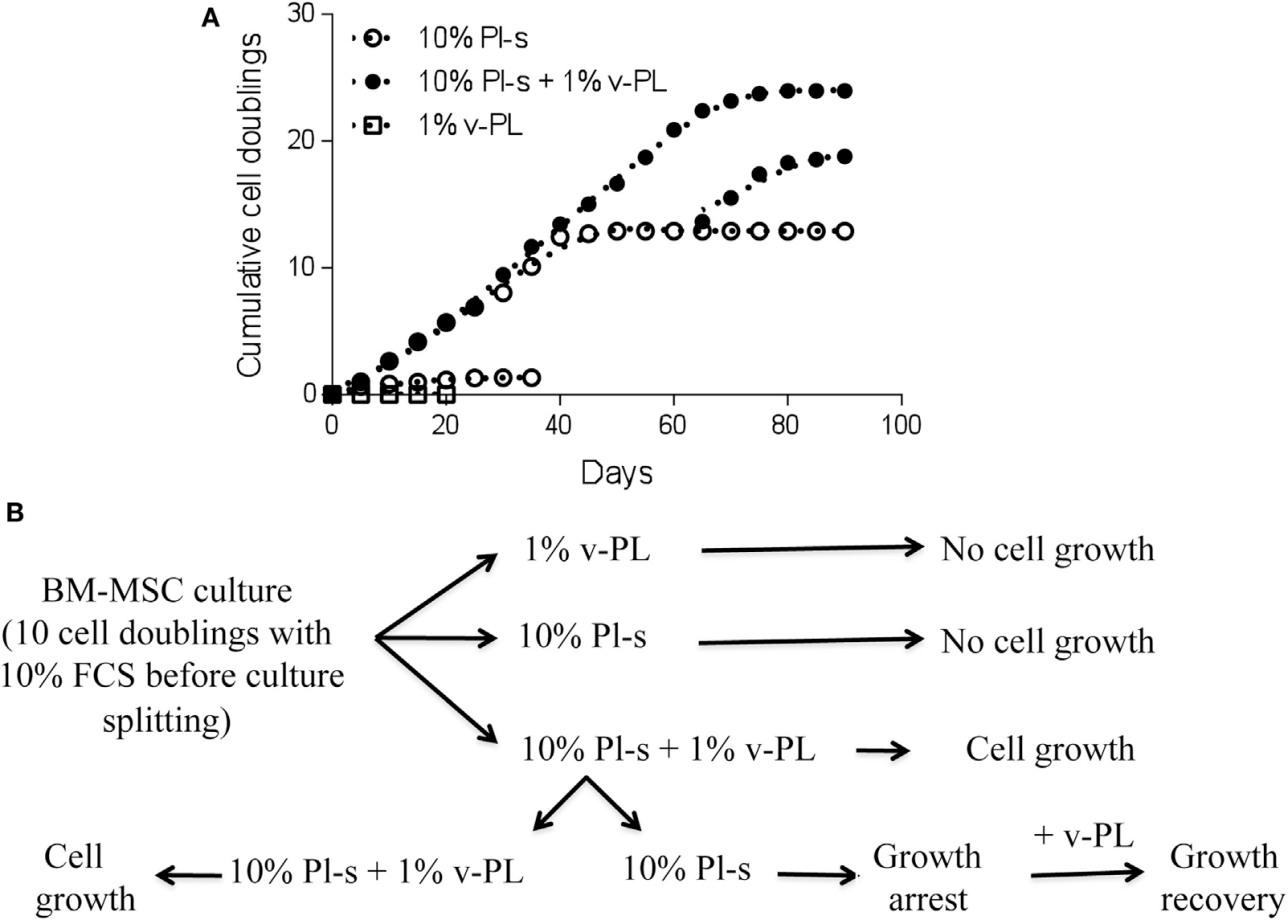
v-PL can rejuvenate a culture of high passage mesenchymal stem cells (MSC). **(A)** A culture of bone marrow-derived mesenchymal stem cells (BM-MSC) previously expanded in the presence of 10% FCS for about 10 population doublings was split in the different culture conditions. After 3 weeks, one part of the cells of the culture in 10% Pl-s + 1% v-PL was transferred in medium supplemented with Pl-s without v-PL. After additional passages, at a time that proliferation was arrested (high passage cells), part of the Pl-s culture was transferred again in medium supplemented with 10% Pl-s + 1% v-PL (restoring in this way, the v-PL mitogenic stimulus). As shown by the graph, 1% v-PL cannot support cell proliferation, but the addition of v-PL to high passage cells, maintained in the presence of Pl-s as the only supplement, rejuvenate the cells that resume proliferation. **(B)** A scheme is reported to summarize the experiment in **(A)**.

The positive effect of v-PL on resting cells was confirmed on a primary culture of BM-MSC (Figure 5). Cells, isolated and expanded in 10% FCS for about 10 population doublings, were split and transferred to different culture conditions: (i) 1% v-PL; (ii) 10% Pl-s; and (iii) 10% Pl-s + 1% v-PL. Both the 1% v-PL only and the 10% Pl-s only culture conditions were not permissive for the cell growth and did not support cell proliferation. However, the combination of the two supplements allowed a good proliferation rate. After about seven doublings, to half of the growing cells, the v-PL was removed, leaving the cells in 10% Pl-s only. The cells maintained in 10% Pl-s + 1% v-PL remained fully viable and continued to grow up to about 25 duplications. On the contrary, in the half culture where the mitogenic stimulus of v-PL was removed, the cells, after a period of adaptation to the new condition, stopped growing and entered a growth arrest status. At this point, half of this culture was maintained in 10% Pl-s only, whereas the other half was transferred again to 10% Pl-s + 1% v-PL (restoring in this way the mitogenic stimulus of the v-PL). When v-PL was provided, high passage growth-arrested MSC resumed proliferation (Figure 5).

### Pl-s versus Plasma

**Figure 6:**
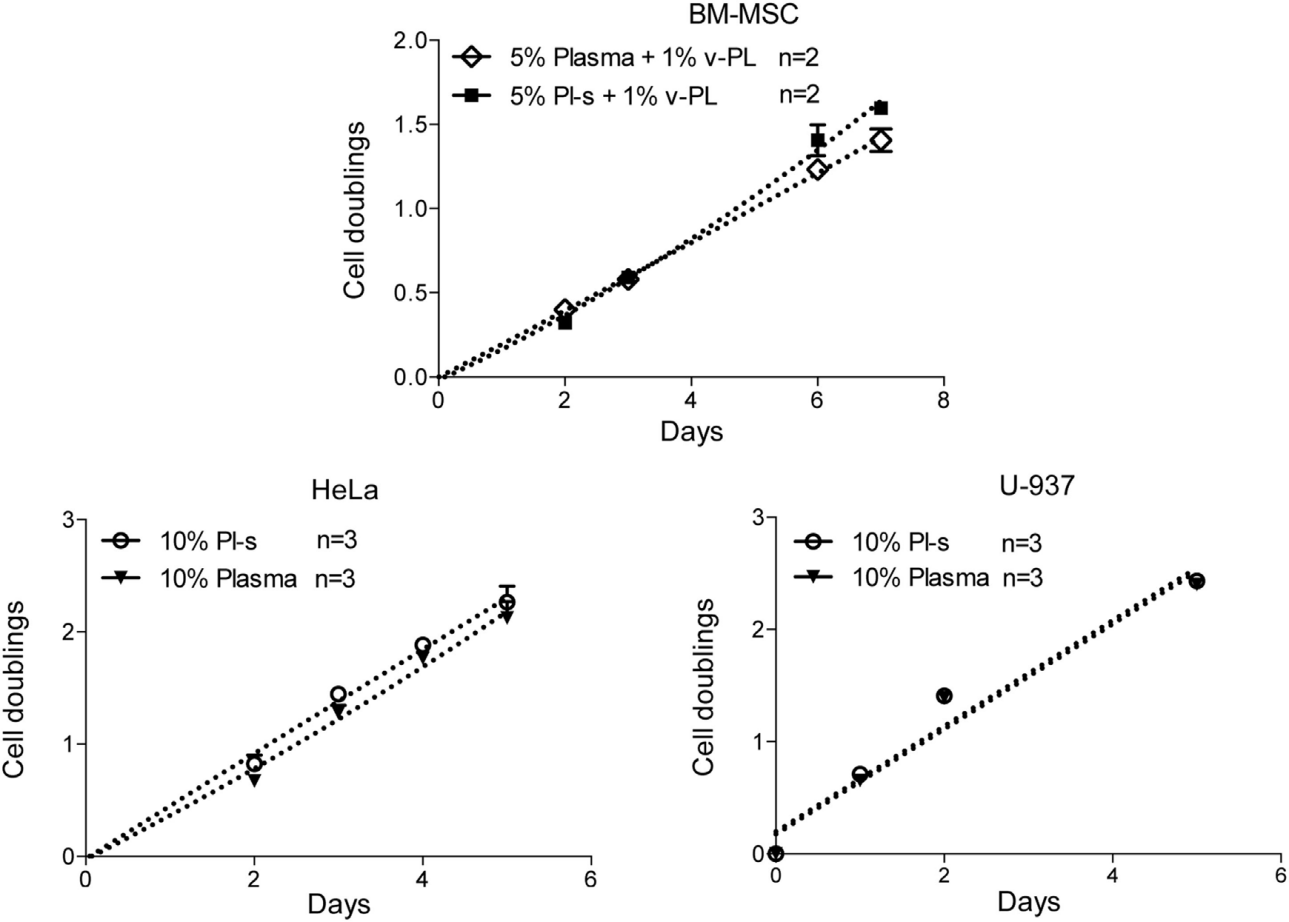
Comparison of Pl-s and plasma as medium supplements. Human primary bone marrow-derived mesenchymal stem cells (BM-MSC) and human cell lines growing either in adhesion (HeLa) or in suspension (U-937) were cultured either with Pl-s or plasma as medium supplements. Cell proliferation was determined by direct cell counting. Number (*n*) of cell cultures used for the experiments is indicated on the graph for each cell type.

A comparison of the efficacy of Pl-s and plasma in sustaining cell proliferation was performed taking advantage of primary cultures of BM-MSC and different cell lines growing either in adhesion (HeLa) or in suspension (U-937) (Figure 6). Plasma- and plasma-derived serum were equally able to sustain cell proliferation. No statistically significant differences were obtained from the analysis as shown in Table 2. However, especially for BM-MSC and HeLa, both cell types growing in adhesion, the Pl-s was slightly more efficient than the plasma from which it was derived.

### Population Doublings of Cells Cultured with the New Supplements versus FCS

**Figure 7:**
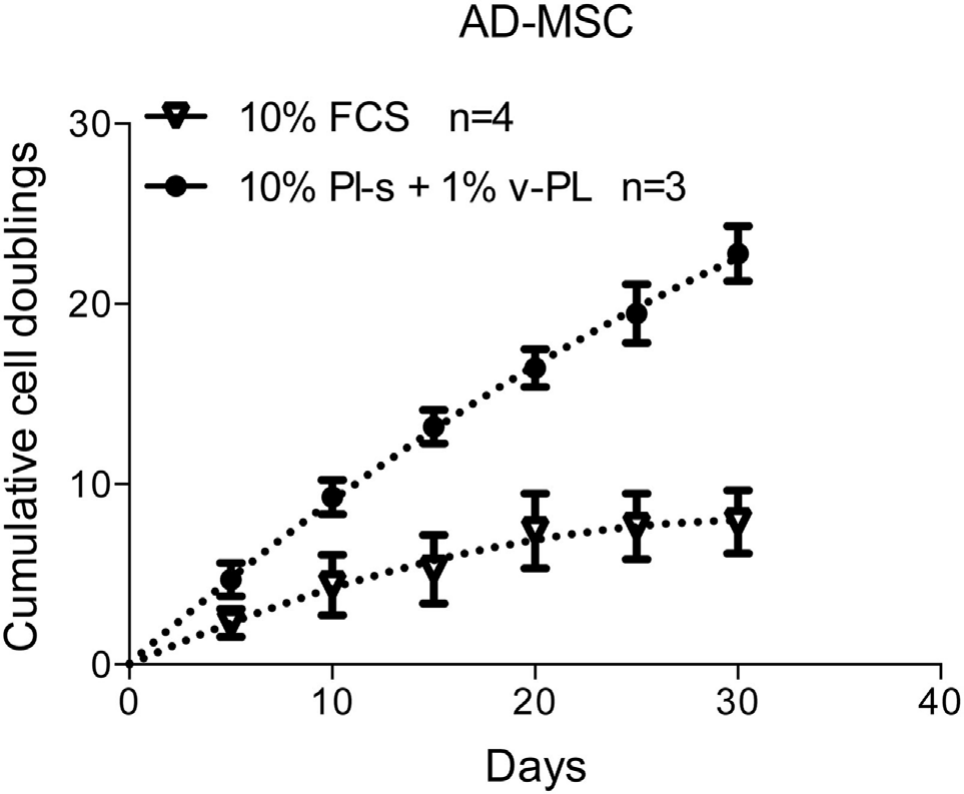
Growth rate of cells cultured with the new supplements. The combined effect of Pl-s and v-PL on cell growth was tested primary cultures of adipose-derived mesenchymal stem cells (AD-MSC) and compared to the control condition where cells were grown with the standard supplement FCS. The proliferation rate was monitored through the evaluation of the cumulative population doublings performed by the two parallel cultures. Number (n) of cell cultures used for the experiments is indicated on the graph for each cell type.

Adipose-derived mesenchymal stem cells were initially isolated and expanded in a medium containing either the standard animal-based 10% FCS or 10% Pl-s + 1% v-PL (Figure 7). Cultures were monitored for about 30 days and the population doublings were calculated. Cells grown with 10% FCS performed a significant lower number of doublings in comparison to the parallel culture maintained in 10% Pl-s + 1%v-PL (6 versus 22 doublings, respectively). The observed difference was statistically significant (Table 3).

**Table 3:** Karyotype stability in cells expanded in the presence of the new culture medium supplements.

Donor	Age (years)	Passage	Doublings	Karyotype
#1	73	2	10	46, XY
#1	73	7	15	46, XY
#2	94	2	11	46, XX
#3	51	2	14	46, XX

### Differentiation Potential of Cells Cultured with the New Supplements

Maintenance of the differentiation potential by the cells expanded in the presence of the new medium supplements was confirmed. Specific *in vitro* assays routinely performed in the lab were adopted. In particular, osteogenic differentiation was tested for BM-MSC and AD-MSC and chondrogenic differentiation for dedifferentiated articular chondrocytes. To note that, in experiments where we compared differentiation of MSC expanded in the presence of the new culture medium supplements and in the standard culture condition, i.e., with FCS as medium additive, we observed a higher extent of osteogenic differentiation in cells cultured with 10% Pl-s + 1%v-PL and a possible shortening of the time before the onset of the osteogenic differentiation.

**Figure 8:**
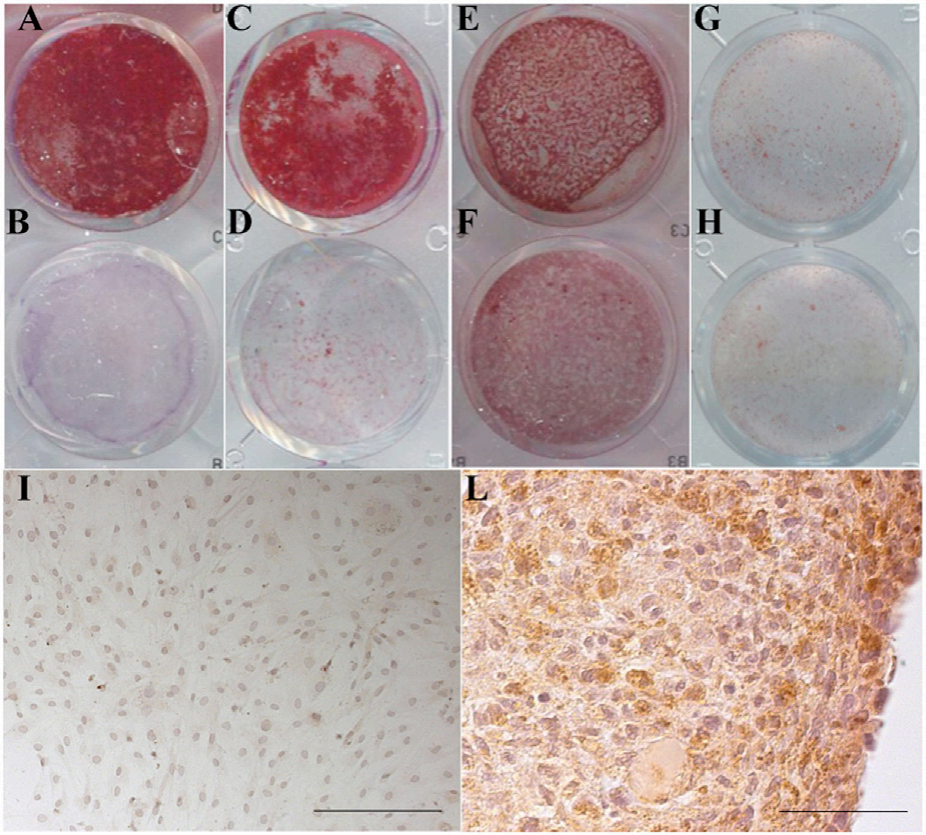
Maintenance of the differentiation potential by cells cultured in the presence of v-PL and Pl-s. **(A,B,E,F)** Primary cultures of adipose-derived mesenchymal stem cells (AD-MSC) (passage 2) cultured in 10%Pl-s + 1%v-PL **(A,B)** or 10% FCS **(E,F)**; **(C,D,G,H)** primary cultures of bone marrow-derived mesenchymal stem cells (BM-MSC) (passage 2) cultured in 10%Pl-s + 1%v-PL **(C,D)** or 10% FCS **(G,H)**; **(A,C,E,G)** cells osteogenically induced; **(B,D,F,H)** not osteogenically induced control cells. **(I,L)** Human articular chondrocytes cultured as adherent dedifferentiated cells [**(I)** scale bar 200 µm] and transferred to suspension cultures [**(L)** scale bar 50 µm] stained with antibodies against αII collagen.

Representative images of the Alizarin Red S staining of MSC and of the type II collagen immunolocalization in the chondrocyte culture are presented in Figure 8. In the stimulated culture condition, it is clearly evident a calcium-enriched matrix and a type II collagen positivity for the MSC and the chondrocytes, respectively.

### Karyotype Stability of Cells Cultured with the New Supplements

**Figure 9:**
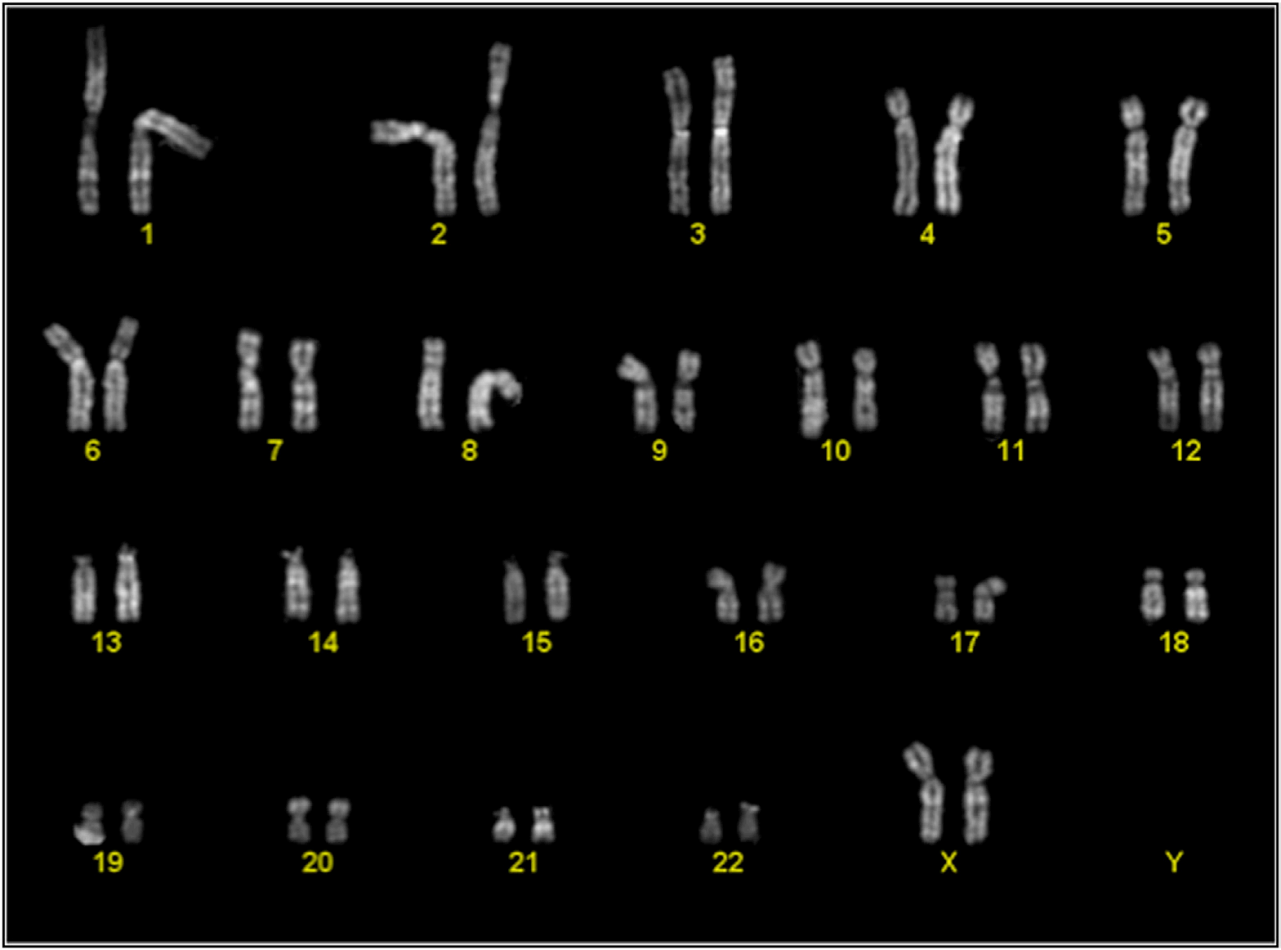
Representative karyotype of a passage 2 bone-marrow derived mesenchymal stem cells culture. Analyses of more than 10 metaphases revealed a normal 46 XX karyotype.

Cytogenetic analysis was performed on passage 2 of 3 cultures of BM-MSC in 10% Pl-s + 1% v-PL, in order to obtain information on the genetic stability of the cells grown in this condition. One of the cultures was also analyzed after a more extensive expansion in the presence of the new supplement (passage 7). The genetic stability was confirmed in all cultures (Figure 9; Table 3).

## Discussion

In almost all studies where *in vitro*-expanded human MSC were adopted for therapeutic purposes, the main cell culture medium supplement was the fetal bovine serum (FBS or FCS). However, in this case, the use of FBS or FCS as medium supplements is undesirable because of the risk of transmitting animal viruses, prion diseases, including the bovine spongiform encephalopathy (BSE) in cows, and proteins that may trigger a xenogeneic immune response. The use of a human autologous serum avoids the zoonotic infection risk, but generation of autologous serum has significant limitations from a manufacturing, regulatory, and quality assurance perspective. Indeed, the large variability observed with sera from different patients deters their use for expansion of cells intended for therapy. Moreover, it is expected that the supply of bovine serum will be highly diminished in the near future years and that research and cell factory labs will be forced to modify their cell culture protocols and to adopt substitutes of FBS. For these reasons, human blood PL supplements are gaining an increasing interest as bovine serum substitutes, especially when cells that eventually will be implanted in a patient are cultured.

Different procedures and protocols have been adopted for the preparation of these PL supplements (Bieback et al., 2009) reviewed in Pawitan (2012) and Shih and Burnouf (2015). In essentially all published protocols, including the recently published one of ours for the production of a two component supplement (Muraglia et al., 2015), the final human platelet-derived additive includes, in addition to growth factors and active molecules present within platelets, also a significant amount of proteins and other molecules component of plasma. To avoid coagulation of the plasma fibrinogen upon addition of the supplement to the culture medium, heparin is normally added as an anticoagulant agent. However, as already mentioned in the introduction, it has been reported by several authors that heparin is capable of interfering with the activity of several growth factors. Hemeda et al. (2013) have described that higher heparin concentrations impaired cellular proliferation in a dose-dependent manner. At high heparin concentrations colony-forming unit frequency and the *in vitro* differentiation toward adipogenic and osteogenic lineages of human BM-MSC were also reduced. Moreover, being of animal origin, the addition of heparin should be discouraged whatever possible, especially when the culture medium is adopted for the expansion of cells intended for cell therapy.

Compared to plasma, proteins and factors involved in the coagulation process are missing in the serum. Conflicting results have been reported on the use of allogenic serum, obtained by conventional blood coagulation or by coagulation of plasma, as culture medium supplement. Initial studies performed more than 10 years ago with human serum revealed the possibility of efficiently isolate and expand human BM-MSC that maintained their differentiation potential (Stute et al., 2004). Shetty et al. (2007) observed an increase of cell proliferation when human BM-MSC cultures were performed in the presence of human serum from whole blood compared to FCS. In the presence of human serum, an enhancement in the adipogenic differentiation of the same cells was observed by Oreffo et al. (1997). Enhancement of MSC differentiation induced by allogenic human serum or autologous plasma was observed also by other authors (Anselme et al., 2002; Yamaguchi et al., 2002). However, Shahdadfar (2005) reported an increased proliferation of human BM-MSC induced by autologous serum compared to FCS, but not by allogenic serum. An increase in the proliferation, but a less effective differentiation of human pre-adipocytes was observed by Koellensperger et al. (2006). Simões et al. (2013) observed an increase in the cell yield when MSC of different origins were cultured with human serum compared to FCS as medium supplement throughout passage 3 to passage 7. Recently, a comparable UC-MSC proliferation was shown in cultures with human serum from two different plasma sources as medium supplement, plasma removed from blood after 24 h from collection and plasma devoid of cryoprecipitate (Dos Santos et al., 2017). However, in most, if not all, of the above-cited papers and similar ones in the literature, a careful determination of the platelet factor concentration in the serum was not done and the adopted protocols not always guaranty the absence of platelets or platelet content as serum contaminants.

Here, we reported the development of two standardized lyophilized cell culture medium supplements: a completely plasma-free platelet lysate (v-PL) obtained from extensively washed platelets and a platelet lysate-free serum (Pl-s) obtained by a plasma cryo-precipitation, to remove the bulk of the fibrinogen molecules, followed by a calcium-mediated coagulation, to remove the residual fibrinogen. Characterization of these two products and investigation on their biological activity when used as cell culture supplements lead us to conclude that v-PL recruited quiescent, and even differentiated or high passage growth-arrested cells back to the cell cycle by activating the cell proliferation machinery (ERK and AKT phosphorylation, Cycline D1 induction, etc.). Instead, v-PL was unable to support cell proliferation unless the plasma or serum components were also present. These observations are in agreement with the interesting results obtained with primary cultures of monkey arterial smooth muscle cells (Ross et al., 1974) and Swiss mouse 3T3 cells, embryo derived cells with a high degree of sensitivity to contact inhibition of growth (Todaro and Green, 1963; Vogel et al., 1978), both types of cells requiring platelet-derived growth factors in association with plasma components to sustain the cell growth, where platelet factors alone did not promote proliferation. Indeed, despite the fact that platelet-derived mitogens were crucial to promote the reentry in the cell cycle (commitment) of confluent cells of early passages and even of late passages of primary cultures, v-PL used as single component did not sustain viability and proliferation of either cell lines or primary cell cultures. On the contrary Pl-s was ineffective in quiescent resting cells, but supported proliferation of the same cells after v-PL treatment and of cells constitutively stimulated as in the case of established cell lines. In agreement with these findings, the combination of the two components was highly effective in supporting proliferation of primary cell cultures at a higher level than FBS in control cultures, whereas the Pl-s added to the medium as a single component sustained the growth of several human cell lines in adhesion or in suspension at a growth rate comparable to the one of FBS control cultures. These results are in line with previous studies, dated back to almost 40 years, where virally transformed cells grew well in plasma-derived serum without the need of exogenous platelet extract, whereas malignant cells of mesodermal origin directly derived from tumors showed a range of dependence on platelet-derived factors (Currie, 1981).

When we determined the efficiency of Pl-s and the plasma from which it was derived with regard to the ability to sustain cell proliferation in cultures of both primary cells and cell lines, we observed a comparable activity in the two medium supplements. However, a slight proliferation advantage was observed in cultures supplemented with the Pl-s. This was especially true for cells growing adherent to the Petri dish compared to cells growing in suspension. This finding suggests that, during the coagulation process, an activation of proteins and factors favoring adherence of proliferating cells is occurring, possibly through enzymatic proteolysis.

The use of the two combined products allowed to establish cell cultures from tissue biopsies or aspirates in complete absence of animal components and to *in vitro* expand different types of cells, intended for cell therapy in humans, maintaining their differentiation potential and without introducing karyotype alterations. These cells include, but are not limited to, MSC derived from bone marrow, adipose or cord blood, and articular chondrocytes. To note is that this not only could consent to perform cell therapies with cells expanded in a safer conditions (absence of animal components in the medium) but, in some cases, could allow to isolate and expand transplantable cells otherwise not achievable with conventional culture medium supplements. This is particularly relevant in case of elderly patients from which conventional culture medium supplements often do not permit to obtain an adequate number of cells.

## Ethics Statement

Ethics Committee of Ospedale Policlinico San Martino has approved the use of samples derived from the human patients as waste material from surgical interventions in the field of orthopedic and plastic surgery and destined for incineration.

## Author Contributions

MM and RC conceived and designed the work; AM, VTN, MN, MM performed the experiments and elaborated data; BD performed the statistical analysis; PS, IB, MF, DC provided samples and reagents, RC and MM participated to the interpretation and critical review of the manuscript. All authors approved the final version of the article.

## Conflict of Interest Statement

RC and MM are shareholders of Biorigen Srl, a spin off company of the University of Genoa, with an interest in Regenerative Medicine. All other authors declare that the research was conducted in the absence of any commercial or financial relationships that could be construed as a potential conflict of interest. The reviewer AB and handling Editor declared their shared affiliation.

## References

[B1] Andress D. L. (1995). Heparin modulates the binding of insulin-like growth factor (IGF) binding protein-5 to a membrane protein in osteoblastic cells. J. Biol. Chem. 270, 28289–28296. 7499327

[B2] Anselme K. Broux O. Noel B. Bouxin B. Bascoulergue G. Dudermel A.-F. (2002). In vitro control of human bone marrow stromal cells for bone tissue engineering. Tissue Eng. 8, 941–953. 10.1089/107632702320934047 12542940

[B3] Ayache S. Panelli M. C. Byrne K. M. Slezak S. Leitman S. F. Marincola F. M. (2006). Comparison of proteomic profiles of serum, plasma, and modified media supplements used for cell culture and expansion. J. Trans. Med. 4, 40. 10.1186/1479-5876-4-40 17020621 PMC1601968

[B4] Balk S. D. Whitfield J. F. Youdale T. Braun A. C. (1973). Roles of calcium, serum, plasma, and folic acid in the control of proliferation of normal and Rous sarcoma virus-infected chicken fibroblasts. Proc. Natl. Acad. Sci. U.S.A. 70, 675–679. 10.1073/pnas.70.3.675 4351800 PMC433333

[B5] Bieback K. Hecker A. Kocaömer A. Lannert H. Schallmoser K. Strunk D. (2009). Human alternatives to fetal bovine serum for the expansion of mesenchymal stromal cells from bone marrow. Stem Cells 27, 2331–2341. 10.1002/stem.139 19544413

[B6] Bottio T. Pittarello G. Bonato R. Fagiolo U. Gerosa G. (2003). Life-threatening anaphylactic shock caused by porcine heparin intravenous infusion during mitral valve repair. J. Thorac. Cardiovasc. Surg. 126, 1194–1195. 10.1016/S0022-5223(03)00813-4 14566272

[B7] Capelli C. Gotti E. Morigi M. Rota C. Weng L. Dazzi F. (2011). Minimally manipulated whole human umbilical cord is a rich source of clinical-grade human mesenchymal stromal cells expanded in human platelet lysate. Cytotherapy 13, 786–801. 10.3109/14653249.2011.563294 21417678

[B8] Cavari S. Ruggiero M. Vannucchi S. (1993). Antiproliferative effects of heparin on normal and transformed NIH/3T3 fibroblasts. Cell Biol. Int. 17, 781–786. 10.1006/cbir.1993.1140 8106075

[B9] Copland I. B. Garcia M. A. Waller E. K. Roback J. D. Galipeau J. (2013). The effect of platelet lysate fibrinogen on the functionality of MSCs in immunotherapy. Biomaterials 34, 7840–7850. 10.1016/j.biomaterials.2013.06.050 23891515

[B10] Currie G. A. (1981). Platelet-derived growth-factor requirements for in vitro proliferation of normal and malignant mesenchymal cells. Br. J. Cancer 43, 335–343. 10.1038/bjc.1981.53 7225284 PMC2010611

[B11] Dos Santos V. T. M. Mizukami A. Orellana M. D. Caruso S. R. da Silva F. B. Traina F. (2017). Characterization of human AB serum for mesenchymal stromal cell expansion. Transfus. Med. Hemother. 44, 11–21. 10.1159/000448196 28275329 PMC5318935

[B12] Gajdusek C. DiCorleto P. Ross R. Schwartz S. M. (1980). An endothelial cell-derived growth factor. J. Cell Biol. 85, 467–472. 10.1083/jcb.85.2.467 7372716 PMC2110613

[B13] Gilotti A. C. Nimlamool W. Pugh R. Slee J. B. Barthol T. C. Miller E. A. (2014). Heparin responses in vascular smooth muscle cells involve cGMP-dependent protein kinase (PKG). J. Cell. Physiol. 229, 2142–2152. 10.1002/jcp.24677 24911927 PMC4149598

[B14] Gospodarowicz D. Ill C. R. (1980). Do plasma and serum have different abilities to promote cell growth? Proc. Natl. Acad. Sci. U.S.A. 77, 2726–2730. 10.1073/pnas.77.5.2726 6930662 PMC349476

[B15] Handschin A. E. Trentz O. A. Hoerstrup S. P. Kock H. J. Wanner G. A. Trentz O. (2005). Effect of low molecular weight heparin (dalteparin) and fondaparinux (Arixtra) on human osteoblasts in vitro. Br. J. Surg. 92, 177–183. 10.1002/bjs.4809 15584059

[B16] Hemeda H. Kalz J. Walenda G. Lohmann M. Wagner W. (2013). Heparin concentration is critical for cell culture with human platelet lysate. Cytotherapy 15, 1174–1181. 10.1016/j.jcyt.2013.05.006 23845186

[B17] Huang Q. Xu T. Wang G.-Y. Huang J.-F. Xia H. Yin R. (2012). Species-specific identification of ruminant components contaminating industrial crude porcine heparin using real-time fluorescent qualitative and quantitative PCR. Anal. Bioanal. Chem. 402, 1625–1634. 10.1007/s00216-011-5590-2 22147273

[B18] Jung J. Moon N. Ahn J.-Y. Oh E.-J. Kim M. Cho C.-S. (2009). Mesenchymal stromal cells expanded in human allogenic cord blood serum display higher self-renewal and enhanced osteogenic potential. Stem Cells Dev. 18, 559–571. 10.1089/scd.2008.0105 18754716

[B19] Khorana A. A. Sahni A. Altland O. D. Francis C. W. (2003). Heparin inhibition of endothelial cell proliferation and organization is dependent on molecular weight. Arterioscler. Thromb. Vasc. Biol. 23, 2110–2115. 10.1161/01.ATV.0000090671.56682.D7 12920044

[B20] Kobayashi T. Watanabe H. Yanagawa T. Tsutsumi S. Kayakabe M. Shinozaki T. (2005). Motility and growth of human bone-marrow mesenchymal stem cells during ex vivo expansion in autologous serum. J. Bone Joint Surg. 87, 1426–1433. 10.1302/0301-620X.87B10.16160 16189322

[B21] Kocaoemer A. Kern S. Klüter H. Bieback K. (2007). Human AB serum and thrombin-activated platelet-rich plasma are suitable alternatives to fetal calf serum for the expansion of mesenchymal stem cells from adipose tissue. Stem Cells 25, 1270–1278. 10.1634/stemcells.2006-0627 17255520

[B22] Koellensperger E. von Heimburg D. Markowicz M. Pallua N. (2006). Human serum from platelet-poor plasma for the culture of primary human preadipocytes. Stem Cells 24, 1218–1225. 10.1634/stemcells.2005-0020 16424400

[B23] Le Blanc K. Samuelsson H. Lönnies L. Sundin M. Ringdén O. (2007). Generation of immunosuppressive mesenchymal stem cells in allogeneic human serum. Transplantation 84, 1055–1059. 10.1097/01.tp.0000285088.44901.ea 17989613

[B24] Mishra-Gorur K. Castellot J. J. (1999). Heparin rapidly and selectively regulates protein tyrosine phosphorylation in vascular smooth muscle cells. J. Cell. Physiol. 178, 205–215. 10.1002/(SICI)1097-4652(199902)178:2<205::AID-JCP10>3.0.CO;2-9 10048585

[B25] Mizuno N. Shiba H. Ozeki Y. Mouri Y. Niitani M. Inui T. (2006). Human autologous serum obtained using a completely closed bag system as a substitute for foetal calf serum in human mesenchymal stem cell cultures. Cell Biol. Int. 30, 521–524. 10.1016/j.cellbi.2006.01.010 16616867

[B26] Mojica-Henshaw M. P. Jacobson P. Morris J. Kelley L. Pierce J. Boyer M. (2013). Serum-converted platelet lysate can substitute for fetal bovine serum in human mesenchymal stromal cell cultures. Cytotherapy 15, 1458–1468. 10.1016/j.jcyt.2013.06.014 24199591

[B27] Muraglia A. Todeschi M. R. Papait A. Poggi A. Spanò R. Strada P. (2015). Combined platelet and plasma derivatives enhance proliferation of stem/progenitor cells maintaining their differentiation potential. Cytotherapy 17, 1793–1806. 10.1016/j.jcyt.2015.09.004 26589754

[B28] Oreffo R. O. Virdi A. S. Triffitt J. T. (1997). Modulation of osteogenesis and adipogenesis by human serum in human bone marrow cultures. Eur. J. Cell Biol. 74, 251–261. 9402473

[B29] Papathanasopoulos A. Kouroupis D. Henshaw K. McGonagle D. Jones E. A. Giannoudis P. V. (2011). Effects of antithrombotic drugs fondaparinux and tinzaparin on in vitro proliferation and osteogenic and chondrogenic differentiation of bone-derived mesenchymal stem cells. J. Orthop. Res. 29, 1327–1335. 10.1002/jor.21405 21432897 PMC3193377

[B30] Pawitan J. A. (2012). Platelet rich plasma in xeno-free stem cell culture: the impact of platelet count and processing method. Curr. Stem Cell Res. Ther. 7, 329–335. 10.2174/157488812802481508 22849700

[B31] Pisciotta A. Riccio M. Carnevale G. Beretti F. Gibellini L. Maraldi T. (2012). Human serum promotes osteogenic differentiation of human dental pulp stem cells in vitro and in vivo. PLoS ONE 7:e50542. 10.1371/journal.pone.0050542 23209773 PMC3510089

[B32] Rauch C. Feifel E. Amann E.-M. Spötl H. P. Schennach H. Pfaller W. (2011). Alternatives to the use of fetal bovine serum: human platelet lysates as a serum substitute in cell culture media. ALTEX 28, 305–316. 10.14573/altex.2011.4.305 22130485

[B33] Ross R. Glomset J. Kariya B. Harker L. (1974). A platelet-dependent serum factor that stimulates the proliferation of arterial smooth muscle cells in vitro. Proc. Natl. Acad. Sci. U.S.A. 71, 1207–1210. 10.1073/pnas.71.4.1207 4208546 PMC388193

[B34] Ross R. Nist C. Kariya B. Rivest M. J. Raines E. Callis J. (1978). Physiological quiescence in plasma-derived serum: influence of platelet-derived growth factor on cell growth in culture. J. Cell. Physiol. 97(3 Pt 2 Suppl. 1), 497–508. 10.1002/jcp.1040970325 103885

[B35] Ruggiu A. Ulivi V. Sanguineti F. Cancedda R. Descalzi F. (2013). The effect of Platelet Lysate on osteoblast proliferation associated with a transient increase of the inflammatory response in bone regeneration. Biomaterials 34, 9318–9330. 10.1016/j.biomaterials.2013.08.018 24012435

[B36] Rutherford R. B. Ross R. (1976). Platelet factors stimulate fibroblasts and smooth muscle cells quiescent in plasma serum to proliferate. J. Cell Biol. 69, 196–203. 10.1083/jcb.69.1.196 1254643 PMC2110973

[B37] Seeger F. H. Rasper T. Fischer A. Muhly-Reinholz M. Hergenreider E. Leistner D. M. (2012). Heparin disrupts the CXCR4/SDF-1 axis and impairs the functional capacity of bone marrow-derived mononuclear cells used for cardiovascular repair. Circ. Res. 111, 854–862. 10.1161/CIRCRESAHA.112.265678 22821930

[B38] Shahdadfar A. Frønsdal K. Haug T. Reinholt F. P. Brinchmann J. E. (2005). In vitro expansion of human mesenchymal stem cells: choice of serum is a determinant of cell proliferation, differentiation, gene expression, and transcriptome stability. Stem Cells 23, 1357–1366. 10.1634/stemcells.2005-0094 16081661

[B39] Shetty P. Bharucha K. Tanavde V. (2007). Human umbilical cord blood serum can replace fetal bovine serum in the culture of mesenchymal stem cells. Cell Biol. Int. 31, 293–298. 10.1016/j.cellbi.2006.11.010 17208468

[B40] Shih D. T.-B. Burnouf T. (2015). Preparation, quality criteria, and properties of human blood platelet lysate supplements for ex vivo stem cell expansion. New Biotechnol. 32, 199–211. 10.1016/j.nbt.2014.06.001 PMC7102808 24929129

[B41] Simões I. N. Boura J. S. dos Santos F. Andrade P. Z. Cardoso C. M. P. Gimble J. M. (2013). Human mesenchymal stem cells from the umbilical cord matrix: successful isolation and ex vivo expansion using serum-/xeno-free culture media. Biotechnol. J. 8, 448–458. 10.1002/biot.201200340 23420807

[B42] Stute N. Holtz K. Bubenheim M. Lange C. Blake F. Zander A. R. (2004). Autologous serum for isolation and expansion of human mesenchymal stem cells for clinical use. Exp. Hematol. 32, 1212–1225. 10.1016/j.exphem.2004.09.003 15588946

[B43] Tanaka Y. Ogasawara T. Asawa Y. Yamaoka H. Nishizawa S. Mori Y. (2008). Growth factor contents of autologous human sera prepared by different production methods and their biological effects on chondrocytes. Cell Biol. Int. 32, 505–514. 10.1016/j.cellbi.2007.12.012 18394935

[B44] Tateishi K. Ando W. Higuchi C. Hart D. A. Hashimoto J. Nakata K. (2008). Comparison of human serum with fetal bovine serum for expansion and differentiation of human synovial MSC: potential feasibility for clinical applications. Cell Trans. 17, 549–557. 10.3727/096368908785096024 18714674

[B45] Tiozzo R. Reggiani D. Cingi M. R. Bianchini P. Osima B. Calandra S. (1991). Effect of heparin derived fractions on the proliferation and protein synthesis of cells in culture. Thromb. Res. 62, 177–188. 10.1016/0049-3848(91)90191-X 1891763

[B46] Todaro G. J. Green H. (1963). Quantitative studies of the growth of mouse embryo cells in culture and their development into established lines. J. Cell Biol. 17, 299–313. 10.1083/jcb.17.2.299 13985244 PMC2106200

[B47] Turnovcova K. Ruzickova K. Vanecek V. Sykova E. Jendelova P. (2009). Properties and growth of human bone marrow mesenchymal stromal cells cultivated in different media. Cytotherapy 11, 874–885. 10.3109/14653240903188947 19903100

[B48] Ulivi V. Tutolo G. Mallein-Gerin F. Daga A. Cancedda R. Cancedda F. D. (2006). A common pathway in differentiation and inflammation: p38 mediates expression of the acute phase SIP24 iron binding lipocalin in chondrocytes. J. Cell. Physiol. 206, 728–737. 10.1002/jcp.20511 16222708

[B49] Vannucchi S. Pasquali F. Fiorelli G. Bianchini P. Ruggiero M. (1990). Effect of heparin on proliferation and signalling in BC3H-1 muscle cells. Evidence for specific binding sites. FEBS Lett. 263, 137–141. 10.1016/0014-5793(90)80723-V 2158901

[B50] Vogel A. Raines E. Kariya B. Rivest M. J. Ross R. (1978). Coordinate control of 3T3 cell proliferation by platelet-derived growth factor and plasma components. Proc. Natl. Acad. Sci. U.S.A. 75, 2810–2814. 10.1073/pnas.75.6.2810 275851 PMC392654

[B51] Yamaguchi M. Hirayama F. Wakamoto S. Fujihara M. Murahashi H. Sato N. (2002). Bone marrow stromal cells prepared using AB serum and bFGF for hematopoietic stem cells expansion. Transfusion 42, 921–927. 10.1046/j.1537-2995.2002.00149.x 12375666

[B52] Zerega B. Cermelli S. Bianco P. Cancedda R. Cancedda F. D. (1999). Parathyroid hormone [PTH(1-34)] and parathyroid hormone-related protein [PTHrP(1-34)] promote reversion of hypertrophic chondrocytes to a prehypertrophic proliferating phenotype and prevent terminal differentiation of osteoblast-like cells. J. Bone Miner. Res. 14, 1281–1289. 10.1359/jbmr.1999.14.8.1281 10457260

